# Trafficking and processing of bacterial proteins by mammalian cells: Insights from chondroitinase ABC

**DOI:** 10.1371/journal.pone.0186759

**Published:** 2017-11-09

**Authors:** Elizabeth Muir, Mansoor Raza, Clare Ellis, Emily Burnside, Fiona Love, Simon Heller, Matthew Elliot, Esther Daniell, Debayan Dasgupta, Nuno Alves, Priscilla Day, James Fawcett, Roger Keynes

**Affiliations:** 1 Department of Physiology, Development and Neuroscience, University of Cambridge, Cambridge, United Kingdom; 2 John Van Geest Centre for Brain Repair, University of Cambridge, Forvie Site, Cambridge, United Kingdom; Aix Marseille University, FRANCE

## Abstract

**Background:**

There is very little reported in the literature about the relationship between modifications of bacterial proteins and their secretion by mammalian cells that synthesize them. We previously reported that the secretion of the bacterial enzyme Chondroitinase ABC by mammalian cells requires the strategic removal of at least three N-glycosylation sites. The aim of this study was to determine if it is possible to enhance the efficacy of the enzyme as a treatment for spinal cord injury by increasing the quantity of enzyme secreted or by altering its cellular location.

**Methodology/Principal findings:**

To determine if the efficiency of enzyme secretion could be further increased, cells were transfected with constructs encoding the gene for chondroitinase ABC modified for expression by mammalian cells; these contained additional modifications of strategic N-glycosylation sites or alternative signal sequences to direct secretion of the enzyme from the cells. We show that while removal of certain specific N-glycosylation sites enhances enzyme secretion, N-glycosylation of at least two other sites, N-856 and N-773, is essential for both production and secretion of active enzyme. Furthermore, we find that the signal sequence directing secretion also influences the quantity of enzyme secreted, and that this varies widely amongst the cell types tested. Last, we find that replacing the 3’UTR on the cDNA encoding Chondroitinase ABC with that of β-actin is sufficient to target the enzyme to the neuronal growth cone when transfected into neurons. This also enhances neurite outgrowth on an inhibitory substrate.

**Conclusion/Significance:**

Some intracellular trafficking pathways are adversely affected by cryptic signals present in the bacterial gene sequence, whilst unexpectedly others are required for efficient secretion of the enzyme. Furthermore, targeting chondroitinase to the neuronal growth cone promotes its ability to increase neurite outgrowth on an inhibitory substrate. These findings are timely in view of the renewed prospects for gene therapy, and of direct relevance to strategies aimed at expressing foreign proteins in mammalian cells, in particular bacterial proteins.

## Introduction

While much is known about expressing mammalian proteins in bacterial cells, little is known about the requirements for passage of a bacterial protein through the secretory pathway of mammalian cells. We have previously shown that strategic removal of at least three N-glycosylation sites is required to achieve secretion of chondroitinase ABC (ChABC), a bacterial enzyme from *Proteus Vulgaris* by mammalian cells [1]. Here we have addressed whether it is possible to increase the efficiency of enzyme secretion by introducing further modifications to the bacterial gene. We removed additional N-glycosylation sites from regions where glycosylation could potentially adversely affect substrate binding. We also assessed the use of alternative leader sequences to direct enzyme secretion from the cells. Further, we evaluated the effect of directing secretion of the enzyme to the neuronal growth cone on neurite outgrowth.

There is currently no effective treatment for promoting regeneration of injured nerves in patients following brain trauma or spinal cord injury. The principal cause of disability is the regenerative failure of mammalian CNS axons, which is due in part to up-regulation of axon growth-inhibitory chondroitin sulphate proteoglycans (CSPGs) in the region of injury [2].

ChABC promotes axon regeneration following CNS injury by removing axon growth-inhibitory CSPGs in the lesion site, and by promoting neural plasticity [3,4]. This latter action, involving formation of new synaptic connections by intact undamaged neurons, has the beneficial consequence of promoting functional recovery. Additionally, we have shown recently that application of the enzyme also promotes the accumulation of anti-inflammatory (M2-like) macrophages at the lesion site [5]. These promote wound resolution and markedly reduce the secondary cavity formation and glial scarring that typically follows injury. ChABC treatment has further been shown to be neuroprotective [6], promoting survival of injured neurons. This robustness of efficacy in experimental SCI has been demonstrated in many injury models and in several mammalian species [4,7,8]. Critically, it is also effective in a rat model of chronic SCI [9], thus greatly extending the number of patients who may potentially benefit from this strategy. This makes it a very strong candidate for treatment of human SCI. Moreover, ChABC also has the potential for wider therapeutic application, since it has recently been shown to improve outcome following peripheral nerve injury [10], and to promote cardiac sympathetic nerve regeneration following experimental myocardial infarction. [11]. Additionally, there are an increasing number of publications describing beneficial results of the enzyme in experimental models of stroke [12,13].

The current approach for treatment of experimental SCI is via multiple intrathecal injections of the bacterial enzyme, and there are two major drawbacks to this approach. First, the enzyme is unstable and consequently loses activity quickly, necessitating multiple injections for efficacy with the attendant risk of further trauma and infection. Second, the molecule is large and therefore diffusion from the injection site to the site of injury is impaired. A gene therapy method of delivery provides a potential solution to these problems. It avoids the need for multiple injections, and since the enzyme is continually produced by cells at the site of injury, the problems of enzyme instability and limited diffusion into deeper regions of the spinal cord are circumvented. Critically, enzyme delivery via a viral vector also enables long-term treatment, as is likely to be required for therapy of chronic SCI. Moreover, because the modified ChABC can be synthesized and secreted by mammalian cells, it enables another approach to the treatment of SCI. Cellular grafts of Schwann cells or olfactory ensheathing cells bridging the site of injury have been shown to improve behavioural outcome following experimental rat SCI (Schwann cells), and recently in human SCI (olfactory ensheathing cells) [14]. The ability to incorporate the ChABC transgene into these cells provides a second, combinatory element of treatment, which we have shown enhances functional efficacy of the transplanted cells in rats [15].

The hurdle we encountered when initiating this gene therapy approach was that active ChABC is not secreted by mammalian cells into which the native bacterial gene has been introduced. We found that this is due to aberrant glycosylation of the bacterial protein by mammalian cells, and identified specific N- glycosylation sites whose removal is required in order to achieve secretion of active enzyme [1]. Here we investigate whether it is possible to further enhance the efficacy of this modified enzyme for clinical application, and we have identified a number of factors, some unexpected, that affect efficiency of ChABC secretion by mammalian cells.

Besides the application of ChABC to treat SCI, other uses for the expression of bacterial protein by mammalian cells include the use of bacterial epitopes for vaccination against bacterial pathogens [16], and for stimulating T cells [17], as well as for inhibition of cancer cell proliferation [18]. A further application is the rapidly expanding field of optogenetics, where bacterial ion channels are used to manipulate membrane potential at the single cell level [19].

Thus, although very poorly researched, the expression of bacterial protein in mammalian cells has potentially a wide range of applications in biology and medicine. Here, using ChABC as an example, we show that some degree of glycosylation is required for secretion of active enzyme, and that the signal sequence employed critically affects the amount of active enzyme secreted. We also show how altering protein localization in neuron can be used to enhance efficacy.

## Materials and methods

### Generation of constructs for transfection

#### Generation of constructs with mutations at additional glycosylation sites

In this study we describe the effect of mutating four previously untested sites located at positions where we considered that N-glycosylation might inhibit substrate binding, but to a lesser extent than those previously described [1]. Constructs were generated by site-directed mutagenesis from the A10 construct (The N-glycosylation sites at positions 282,338,345 &751 are mutated N to Q. The N-glycosylation site N515 is mutated at the+2position S517 to A. [1]).This construct was selected as it contains the largest number of enzyme activity-enhancing mutations. Mutants containing single or double mutations were generated. These were tested for glycosylation by *in vitro* transcription/translation in the presence of canine microsomes as described below *In vitro* transcription/translation was also used to confirm enzyme activity as described in Muir et al [1]. These reactions contained lysate and plasmid but no label or canine microsomes. Enzyme activity was assessed using a Morgan-Elson reaction.

The site-directed mutagenesis primer sequences are as follows:

N-836 to D gcagaaaaagtaGatgtaagtcgccaacaCcaggtttcag
N-856 to Q gacagaaggaCaGtttagctcggcatggatcgatcacagg
N-963 to Q gtcagtgcagttacacctgatCtaCaGatgactcgcc
N-773 to Q ggtagcaatatTCaGagtagtgataaaaataaaaatgttgaaacg

The glycosylation sites N-836 &N-963 were shown not to be glycosylated (*in vitro* transcription/translation in the presence of canine microsomes) and were not investigated further.

#### Generation of constructs with different signal sequences

The signal sequences for human prolactin [20], native bacterial ChABC, Ig kappa (Invitrogen psec Tag leader) and mouse MMP-2 were synthesised and ligated to the modified coding sequence of ChABC [3] using the Spe1 site at the 5’ end of the coding region. The signal sequence for rat GDNF was attached to the modified ChABC coding sequence using fusion PCR [21]. All constructs were confirmed by sequencing. The sequences of the junctions are given in S1 Table. Signal sequences: Juction sequences between signal and ChABC gene.The functionality of these constructs was determined by transfection into four different cell types followed by assessment of the amounts of enzyme secreted into the culture medium using western blots to measure digestion products of the enzyme and/or the enzyme levels present, as described in Muir et al. 2010[1].[22][23]A construct encoding GFP was used to monitor transfection efficiencies.

#### Generation of constructs to direct ChABC to neuronal growth cones

1. Addition of the human β-actin 3’UTR

Site-directed mutagenesis was performed to introduce an EcoRV site 3’ to the bovine growth hormone polyadenylation signal of the pcDNA 3.1(-) ChABC construct [3]This new construct also contains an mcherry tag at the 3’end [22] which allows identification of successfully transfected cells. It has an MMP-2 signal sequence, all the mutations identified that enhance enzyme activity/secretion (N-282, -338, -345, S-515 and N-675 glycosylation sites removed) and codons optimised for expression in mammalian cells. The construct was cut with BamHI/EcoRV and dephosphorylated using shrimp intestinal phosphatase, prior to agarose gel electrophoresis, to remove the bovine growth hormone polyA signal. The β-actin 3’UTR was cut out of pCLNCX β-actin GFP, (a generous gift from Profs W Harris and C Holt). This construct contains the full-length coding sequence for human β-actin fused to GFP. PCR was carried out on the gel-purified fragment with primers containing a BamHI site at the 5’ end and a 3’ primer containing an extension at the 3’ end to allow cutting with Cla1. The PCR product was cut with Cla1, blunted with T4 DNA polymerase, cut with BamHI and then purified by agarose gel electrophoresis and ligated to pcDNA3.1 ChABC mcherry [22] cut with BamHI/EcoRV. Recombinant clones were identified by restriction digest (StyI) and confirmed by sequencing. PCR was carried out using KOD hot start polymerase (Novagen) according to the manufacturer’s instructions for the enzyme. Annealing was carried out at 52°C for one cycle and 57°C for the subsequent 30 cycles, extension was 20 sec for all cycles.

Primers

        PCR

        Forward: 5’CTAAGGATCCAATCAACC 3’

                        BamH1

        rev primer:5’ ATTTTATCGATCAGGCGG 3’

                        Cla1

        Site- directed mutagenesis primer to introduce EcoRV site.

        GGGCTCTAGGGGATATCCCCACGCG

                        EcoRV

The functionality of this new construct was confirmed by transfection into HEK cells followed by a Morgan-Elson enzyme assay to measure the levels of enzyme secreted into the medium.

2. Control plasmids

Control plasmids for these transfections were pCLNCX (Imgenex.com), coding for GFP with human β-actin 3’UTR (positive control), and pcDNA 3.1 ChABC mcherry fusion non-targeted construct (negative control).

### In vitro transcription/translation

Briefly, *in vitro* transcription/translation reactions were carried out with rabbit reticulocyte lysate in a coupled reaction with T7 polymerase using the TNT Quick Coupled transcription/translation kit (Promega). Each 25μl reaction contained 1μg of plasmid, 1μl of canine microsomes (Promega) and 1μl^35^S-methionine^.^ Samples were incubated at 30°C for 90 min. 5μl samples were separated on 8% reducing Tris/glycine gels (Invitrogen), fixed in methanol/acetic acid, and incubated in Amplify (Amersham) for 30min prior to drying. The dried gels were then exposed toX-ray film for 2h.

### Morgan-Elson enzyme assay

This enzyme assay measures ChABC activity by the N-acetylation of product disaccharides and subsequent reaction to give a coloured product. The reaction contains 100μl of 40mM sodium acetate, 40mM Tris-Cl pH8.0, 10mg/ml chondroitin-6-sulphate (Sigma), mixed with 20μl of enzyme sample. *P*.*Vulgaris* ChABC (Sigma) was used as a standard. The reaction was incubated at 37°C for 20min, then stopped by boiling for 1min. Potassium borate solution (0.8M, pH9.1, 100μl) was added and the mixture boiled for 7min. It was then chilled on ice and centrifuged in a microfuge at 13,000rpm for 10 mins. 1ml of glacial acetic acid was added to the supernatant and mixed before centrifugation for a further 20min. To 1ml of supernatant, 0.4ml of Morgan-Elson Reagent (10g paradimethylamino-benzaldehyde in 100ml of glacial acetic acid containing 12.5% concentrated HCl) was added and incubated at 37°C for 20min. Product was measured by absorbance at 550nm.

### Cell culture

#### Cell line propagation

The Neu7 cell line (23) is derived from cortical astrocytes taken from the cerebral cortex of rat pups on the day of birth. The SCTM41 cell line [24] is derived from rat sciatic nerve taken on post-natal day 2. Neu7 cells were grown in Dulbecco’s modified Eagle’s medium (DMEM) containing 10% fetal bovine serum, 10% horse serum and supplemented with penicillin and streptomycin. Cos7 cells the SCTM-41 Schwann cell line and SH-SY-5Y neuroblastoma cells (ATTC CRL-2266) were cultured in DMEM containing 10% FCS supplemented with standard concentrations of penicillin and streptomycin. The NG108 hybridoma cell line (ATCC HB-12317^TM^) was cultured in DMEM, containing 10% fetal bovine serum and supplemented with 1%HAT (hypoxanthine,aminopterine,thymidine) and antibiotics as described in [25].

#### Primary cell cultures

Pregnant females were supplied by Charles River Laboratories, Margate, UK. The rats were housed under a 12h light/dark cycle with *ad libitum* access to food and water. Euthanasia was carried out by decapitation. Decapitation of rat pups is considered a regulated procedure under the Animals Scientific Procedures Act 1986 and as such was authorised under a project License (70/7920, 19b6 “Acquisition of Tissue”) which was ethically reviewed by the University of Cambridge Animal Welfare &Ethical Review Body (AWERB) prior to submission and subsequent approval by the Secretary of State. The University of Cambridge holds an Establishment License (80/2802, X81BD37B1) and is committed to animal welfare, with all animal facilities designated under the above Act. E18 cortical neurons were obtained via appropriate Schedule 1 method at this establishment.

Primary cell cultures were isolated from the tissue by dissociation of the tissue with 0.1% trypsin (Invitrogen) for 15 min at 37°C. The cell suspension was then washed Hanks balanced salt solution (HBSS) prior to cell plating.Primary glial cells are from the cortex of 1–2 day old Wistar rat pups. These cells were cultured in DMEM with 10% fetal bovine serum supplemented with penicillin and streptomycin. They were passaged after one week (leaving a culture consisting mainly of astrocytes), and used for transfection several days later. The primary cultures of cortical neurons are from embryonic day 18 rat pups. These neuronal cultures were grown in in Neurobasal medium (Invitrogen) supplemented with B27 (Invitrogen), glutamic acid (Sigma), penicillin and streptomycin.

### Transfection

#### Transfection of Neu7, SCTM41, COS7 cells and mixed glial cultures

Transfections were performed in 25-cm^2^ flasks with the cells ~60–70% confluent, using Fugene transfection reagent (Roche),5μg plasmid and 0.5μg of pAdvantage vector (Promega) to increase the translation efficiency of the chondroitinase gene. Transfections were performed according to the manufacturer’s instructions. 24h post-transfection, the medium was replaced with Neu7 conditioned medium as a source of CSPGs. This CSPG-containing medium was incubated with the transfected cells for 24h to allow digestion of the CSPGs by any ChABC produced as a result of the transfections. It was then harvested, centrifuged to remove detached cells, and concentrated 10- fold in a centricon-50 unit (Millipore). Protease inhibitor cocktail (Sigma) was added and the medium frozen for subsequent analysis by western blot. In every round of transfection, transfection efficiency was determined using a plasmid encoding GFP

#### Transfection of NG108 and SH-SY5Y cells

NG108 cells were transfected with targeted or non-targeted GFP or targeted or non-targeted ChABC as described for the SH-SY5Y cells below. They were then differentiated by reducing the serum to 1% and plated onto poly-L-Lysine/laminin-coated slides as described in [25]. The cellular location of GFP or ChABC was visualized directly using fluorescent microscopy in the green (GFP) or red (ChABC, detects the mcherry tag) channel. Detailed analysis of the constructs designed to direct ChABC to the neuronal growth cone, was conducted using the SH-SY5Y neuronal cell line [25].For this study, cells were split, the day before transfection, to achieve 50% confluence on the day of transfection. They were then transfected with 5μg of DNA construct using Fugene transfection reagent (Roche) supplemented with 0.5μg of pAdvantage vector to enhance translation [1]. 24 hours post- transfection, cells were dissociated and suspended in DMEM containing 1% FCS, and 10^-6^M retinoic acid to promote differentiation of SH-SY-5Y cells into cells with morphological and biochemical characteristics of mature neurons [26]. They were then plated onto 4-well slides at a density of 1.7x10^5^ cells/well in DMEM. Slides were pre-coated with poly- l-lysine, (100 μg/ml) then with laminin (10 μg /ml) or a mixture of laminin (10μg/ml) and CSA (50μg/ml) to produce an inhibitory environment for neurite out-growth. CSA, a kind gift from Dr. Wen-gang Chai, Imperial College London, is known to be inhibitory to axon outgrowth [27]. All slides were incubated overnight at 37°C and washed with DMEM prior to plating the cells. Cells were allowed to attach, and the following day antibody staining was performed. The concentration of CSA required to inhibit neurite outgrowth in this cell culture system was determined by coating the slides with a laminin/CSA mixture containing a constant amount of laminin and CSA concentrations between 10ng/ml and 50 μg /ml. Non-transfected cells were plated onto these slides and neurite lengths were measured and statistically compared to those obtained on laminin alone.

#### Transfection of primary cortical neurons

Neurons were transfected by electroporation in serum and antibiotic-free medium according to the manufacturer’s instructions using a microporator (Thermofisher) and neon transfection kit (Thermofisher). The conditions optimal for cortical neuron transfection were determined to be 1500V, 10msec, 3 pulses. Transfected cells were plated onto coverslips coated with poly-L-lysine, laminin (10μg/ml) and CSA (50μg/ml), then cultured overnight in serum/antibiotic-free medium. The next day the medium was replaced with supplemented neurobasal medium as described above. 48h post-transfection the neurons were fixed and co-stained for the mcherry tag and β-tubulin as described below.

Neu7, SCTM41, COS7 and NG-108 cells were a gift from Professor James Fawcett. SH-SY5Y cells were a gift from Professor Jenifer Morton, both from the University of Cambridge.

### Production of medium containing CSPGs

(Required for analysis of the constructs containing the new glycosylation mutations or the different signal sequences).

To produce medium containing CSPGs (a substrate for ChABC), Neu7 cells which produce CSPGs (23) were grown in DMEM containing 10% fetal calf serum and 10% horse serum. When the cells had just reached confluence the medium was replaced with DMEM supplemented with ITS^+3^.The medium was collected after 48h and stored at -80°C until required.

### Antibody staining

Cells (SH-SY5Y neurons and primary cortical neurons) were washed (2x5mins in PBS) then fixed in 4% PFA for 15min at room temperature. Following fixation the cells were washed (3x5min in PBS), permeabilised in 0.2% Triton X-100 for 5 minutes then washed (3x5min in PBS). They were then blocked, first with Image-iT FX Signal Enhancer (Invitrogen) for 30 minutes in a humid environment and then with blocking buffer (0.3% Triton X-100, 10% goat serum in PBS) for 2 hours at RT. They were then stained with rabbit polyclonal anti-dsRed antibody (Clontech; 1:500). This stains the mCherry tag present on the ChABC constructs and allows identification of successfully transfected cells. Co-staining with a mouse monoclonal antibody to beta tubulin III (Sigma, 1:1000) allowed visualisation of neurites. Both antibodies were applied in blocking buffer overnight at 4°C. Following 4x 5min washes with PBS, slides were incubated for 1 hour at room temperature in the secondary antibodies: AlexaFluor 488 goat anti-mouse IgG and AlexaFluor 568 donkey anti-rabbit IgG (Invitrogen; both 1:2000). Following 3x 5min washes with PBS, they were mounted with Prolong Gold anti-fade reagent (Invitrogen) and coverslipped. Cells transfected with the GFP constructs were stained as above except the primary antibody was rabbit anti-GFP (1:400).

### Western blots

Sodium dodecyl sulphate polyacrylamide gel electrophoresis (SDS-PAGE) and Western blotting were carried out and the blots probed with antibodies to NG2 (Santa Cruz diluted 1:1000), 1B5 stub antibody (Seikagaku, product Delta Di-0S,diluted 1:250), or anti-ChABC (Acorda Therapeutics Inc, diluted 1:2000) as described in Muir et. al.[1]. Briefly, for detection of chondroitin sulphate proteoglycans or ChABC, 50μl samples of conditioned media from each transfection were separated by SDS-PAGE (6% acrylamide gel, non-reducing conditions), and electroblotted in a Transblot Semi-dry Transfer Cell blotter (Biorad) to Hybond-ECL membrane. Membranes were incubated in 2%ECL Advanced Blocking Agent (Amersham) in Tris-buffered saline with 0.1%Tween-20 (TBS-T) at room temperature for 4h, then incubated with primary antibody in blocking solution overnight at 4°C. Membranes were washed with TBS-T before incubation with secondary antibody (peroxidase-labeled anti-mouse or anti-rabbit IgG, diluted 1:10,000 to 1:30,000 in blocking solution) for 1h at room temperature. Membranes were washed with TBS-T before reaction with ECL chemiluminescence detection reagent and visualized using Hyperfilm (Amersham).

### Fluorescence microscopy and image analysis

Images were captured on a Zeiss Axioplan microscope, under red or green fluorescent light using a digital camera (QImaging) and the imaging software QCapture Pro6.1, which allowed the acquisition exposures to be kept constant for all images within a set. All image analysis was performed using ImageJ (NIH). A segmented line was used to measure neurite lengths. Measured lengths were determined from the image using a 40x objective. Growth cones were identified based on morphology and the average pixel intensity measurements of the whole cell and at the growth cone were used to demonstrate growth cone targeting (arbitrary intensity units (AIU), ImageJ). Low-pass cut-off for fluorescence was set at 20 AIU. Average intensity at the growth cone divided by average whole neuron intensity gave a growth cone intensity index.

### Data analysis and statistics

Data were statistically analysed using the IBM SPSS statistics software (version 21.0; IBM Co, Armonk, NY, USA). For growth cone targeting analysis, histograms and the neurite length analysis distributions showed that data were not normally distributed. This was confirmed using the Anderson Darling normality test (P<0.0005, n = 67). Therefore the non-parametric Mann-Whitney U test (MWU) was used to determine differences between groups, being a suitable test for this dataset as it corrects for ties. This was necessary due to the high number of zero values recorded in the datasets from the growth cone targeting experiments. A Fisher’s exact test confirmed a significantly large number of zero values for the non-targeted ChABC construct (P<0.001).

## Results

### Glycosylation

We have shown previously that active ChABC can be secreted from mammalian cells by removal of the N-glycosylation sites at positions N-282–338–345, and 515, and that mutation of N-675 further increases the activity of enzyme secreted into the medium, as determined by the extent of digestion of CSPGs present in the medium of cells transfected with the different constructs [1]. Unmodified bacterial ChABC contains, in total, 17 potential N-glycosylation sites. The five sites above, and N-751, were identified by 3D modelling of the ChABC molecule using the program Rasmol, as places where the addition of sugar chains to the molecule would most likely influence enzyme activity, by blocking the active site or sterically hindering substrate binding. The site at N-751 was found not to be glycosylated by *in vitro* transcription/ translation in the presence of canine microsomes.

**Fig 1:**
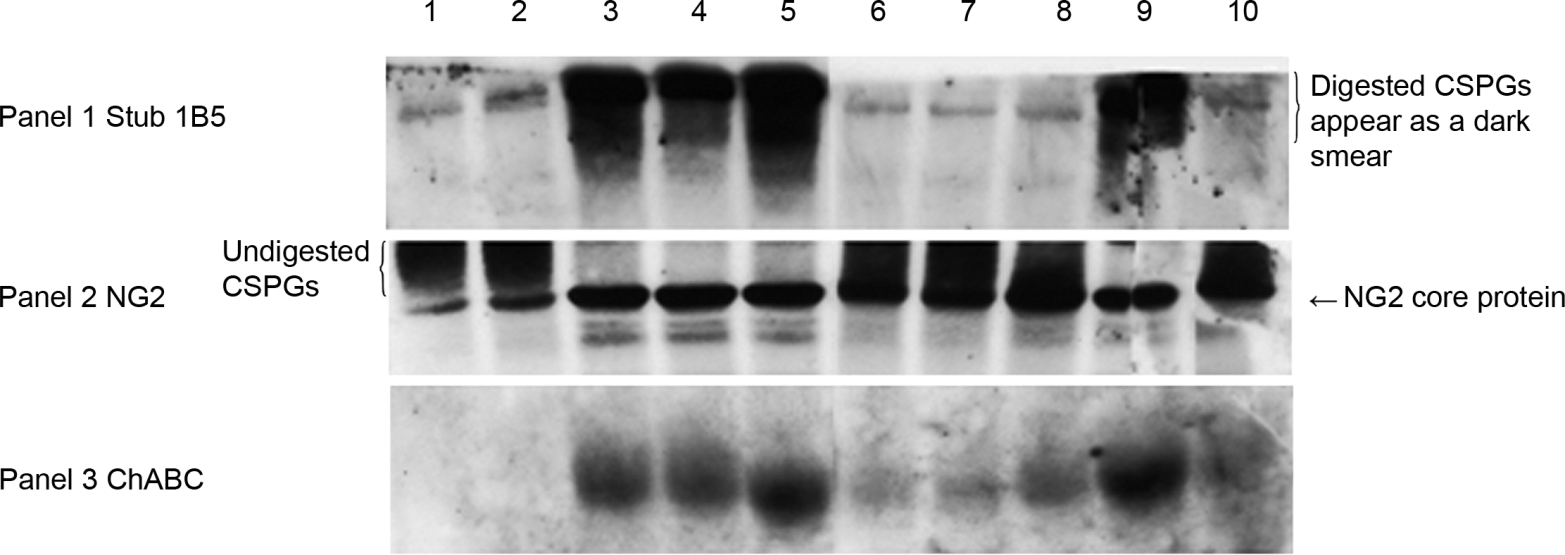
Effect of site-specific glycosylation on chondroitinase secretion.

Here we investigated four further sites predicted to be glycosylated and which could potentially affect enzyme activity by interfering with substrate binding. All mutations produced active enzyme *in vitro* as assessed by *in vitro* transcription /translation. The sites at N-836 and N-963 were found not to be glycosylated. However, mutations of the remaining two sites tested led to some unexpected findings. Mutations at positions N-856 and N-773 both attenuated secretion of the enzyme and were summative (Fig 1). Thus while mutations at some specific N-glycosylation sites are required to achieve secretion of active enzyme, the removal of at least two others is deleterious to both production and secretion of active enzyme.

Cos 7 cells were transfected with constructs containing mutations at different glycosylation sites and medium containing chondroitin sulphate proteoglycans (CSPGs) applied. Western blot of the conditioned medium probed x3. Antibody 1B5 detects the digestion products of ChABC after removal of the glycosaminoglycan chains (GAG chains) exposes the stub epitope. Enzyme activity is detected by appearance of a dark smear. The antibody to NG2 detects both digested and undigested CSPGs. ChABC digestion results in a reduction of the high molecular weight smear as the GAG chains are removed. In the case of complete digestion a single band consisting of the core protein is observed. Lanes 1&2 GFP, Lanes 3&4 starting clone, N-glycosylation sites N-282, N-338, N-345 & N-515 removed. This construct contains an MMP-2 signal sequence and previously identified mutations that enhance enzyme activity and/or secretion. [15]. Lanes 5–10 show the effect of removal of additional glycosylation sites. Removal of the glycosylation site at N-675, lanes 5&9, increases the amount of enzyme secreted. In contrast, removal of another glycosylation site at N-856 markedly reduces the amount of enzyme secreted, lanes 8&10, an effect that is further enhanced by mutation of the glycosylation site at N-773, lanes 6&7. Reduction in the quantity of enzyme secreted (panel 3) is mirrored by a decrease in enzyme activity (panels 1&2). Each experiment was performed twice in duplicate.

### Signal sequences for secretion

Eukaryotic proteins destined for the extracellular environment of the secretory pathway are first translocated across the endoplasmic reticulum (ER) membrane. Their initial segregation to the ER requires a signal sequence encoded at the N-terminus which is recognised co-translationally by the signal recognition particle (SRP). This complex is subsequently targeted, via an interaction with the SRP receptor to an ER translocon which facilitates insertion of the nascent chain into the translocation channel, after which further protein synthesis ensues. It was originally thought that these signal sequences are functionally equivalent and mostly interchangeable. However, further research has demonstrated large differences in the efficiencies between different signal sequences in their ability to promote translocation of the nascent peptide into the ER [20,28]. Although the efficiency of translocation can be influenced by the mature domain of the protein that immediately follows the signal sequence, in the majority of cases it is determined by the properties of the signal sequence itself [29]. The selection of an appropriate signal sequence can be crucial to obtaining high levels of protein expression, with reports of 4-fold differences in the levels of expression obtained with different signal sequences [30]. Furthermore, the efficiency of a particular signal sequence can also vary with cell type [29]. We therefore compared the efficiencies of five different signal sequences in directing secretion of ChABC in a range of cell types.

We found that the signal sequences for secretion are both cell-type-dependent and critical to the efficiency with which ChABC is secreted.

**Fig 2:**
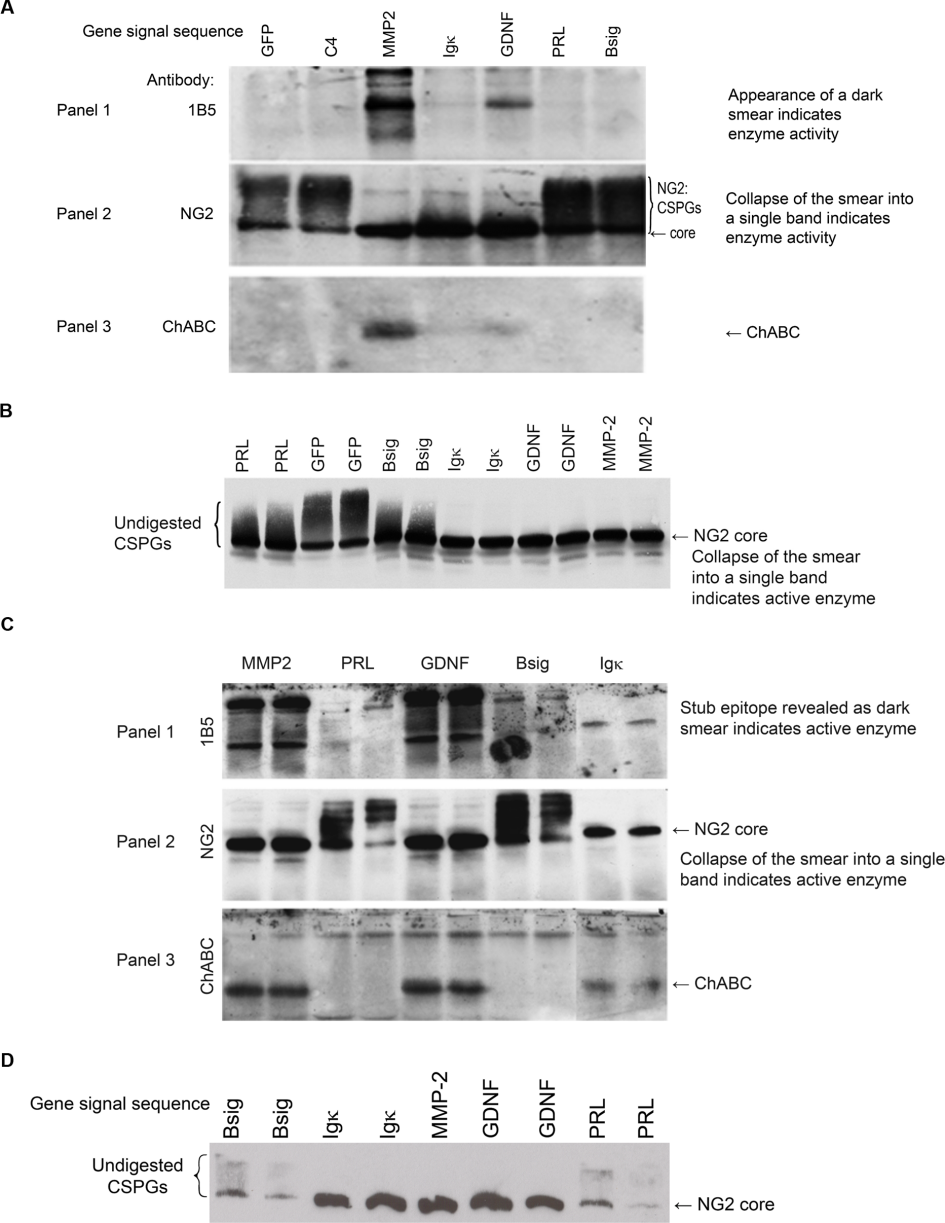
Effect of different signal (leader) sequences on the amount of chondroitinase secreted by different cell types: Fig 2a mixed glial cell culture, Fig 2b SCTM 41 Schwann cells, Fig 2c Cos7 cells, Fig 2d Neu7 cells.

The signal sequence from mouse metalloprotease-2 (MMP-2) functioned efficiently in all the cell types we tested. It directed secretion of active enzyme from primary cultures of glial cells (mostly astrocytes) isolated from rat brain (Fig 2A). A strong signal in the MMP-2 lane is detected with the 1B5 antibody indicating active enzyme has been secreted into the medium and removed the sugar

(Glycosaminoglycan, GAG) chains from the CSPGs present, thus exposing the epitope recognised by the 1B5 antibody. The medium contains a mixture of CSPGs of varying molecular weights which are thus detected as a broad diffuse bands by the IB5 antibody. The antibody to NG2 detects a single proteoglycan. It recognises both intact NG2 with its sugar (GAG) chains attached and the core protein (without sugar chains) which remains following digestion with ChABC. A single band is detected in the MMP-2 lane with the NG2 antibody. This is the core protein of NG2 which remains following complete removal of the GAG chains from NG2 and confirms the result obtained with the 1B5 antibody. A strong signal for ChABC protein is also seen in panel 3, showing that the enzyme has been secreted into the medium. The MMP-2 signal sequence also functions well in the glial cell (Schwann cells and astrocyte) lines tested (Fig 2B and 2D). In both cell types the sugar chains are removed from the core protein of NG2 which appears as a single band. It also functioned well in Cos7 cells (Fig 2C). An intense-staining smear in the MMP-2 lane is seen following staining with the 1B5 antibody, Fig 2C, panel 1, the NG2 is seen as a single band corresponding to the core protein, Fig 2C, panel 2 and ChABC is detected with the anti-ChABC antibody Fig 2C, (panel 3).The GDNF signal sequence functioned efficiently in the SCTM41 (Schwann cell) (Fig 2B), and Neu7 (astrocyte)(Fig 2D) cell lines as the NG2 protein is seen as a single band. It also functioned in Cos7 cells (Fig 2C). A single band corresponding to the NG2 core protein (Fig 2C, panel 2) can be seen in the GDNF lane and an intense staining smear is observed following staining with the 1B5 antibody (Fig 2C, panel 1). Fig 2C, panel 3 shows the presence of ChABC in the medium. However the GDNF signal sequence functioned less well in the mixed glial cultures (Fig 2A). Weak staining is observed after 1B5 and anti-ChABC-staining (Fig 2A, panels 1&3) and there is a smear above the core protein after staining with the anti-NG2 antibody (Fig 2A, panel 2), consistent with the presence of undigested sugar chains. We also found that the signal sequence of the Ig kappa chain, commonly used in commercial plasmids to direct secretion of protein from cells, functioned as well as the MMP-2 signal sequence in Schwann cell (Fig 2B) and Neu7 astrocyte(Fig 2D) cell lines. This was demonstrated by the complete removal of the GAG chain smear above the core protein (NG2 staining; Fig 2B & 2D). However, it was less efficient than the MMP-2 signal sequence in Cos7 cells (Fig 2C). The signals seen after 1B5 and anti-ChABC are much weaker than those obtained with the same construct containing the MMP-2 signal sequence. Moreover, the Ig kappa signal sequence functioned poorly in cultures of mixed glial cells (Fig 2A). A faint signal is visible following staining with 1B5 and anti-ChABC antibodies, Fig 2A, panels 1&3, and a smear representing undigested GAG chains is evident above the core protein after NG2 staining, Fig 2A, panel 2. Furthermore, the prolactin signal sequence and native bacterial signal sequence of the ChABC gene were ineffective at directing secretion of ChABC in all cell types tested, with the possible exception of the Schwann cell line, where a slight decrease in the GAG smear above NG2 core protein was seen with the construct containing the prolactin signal sequence (Fig 2B).

These results show that while the MMP-2, GDNF and Ig kappa signal sequences can function with the ChABC coding sequence, their efficiency varies and is cell type-dependent. Where differences are observed, the efficiency is MMP-2>GDNF>Ig kappa. Also of note is that the ChABC bands on western blots of medium from cells transfected with the constructs containing the signal sequences Ig kappa and GDNF migrate slightly slower than those transfected with the construct containing the MMP-2 signal. This may correspond to an extra site being glycosylated in these proteins as a result of earlier cleavage of these signals compared with proteins containing the MMP-2 signal [31].

Cells were transfected with constructs containing the modified ChABC gene (N-282, N-338, N-345, &N-515 removed [15]) and one of several different signal sequences directing cell secretion of the enzyme. Western blots of CSPG-containing medium from transfected cells were probed with antibodies to ChABC to detect the quantities of enzyme secreted, and/or with antibodies to determine the degree of CSPG digestion occurring as a measure of enzyme activity (1B5, NG2). Antibody 1B5 detects the digestion products of ChABC after removal of the glycosaminoglycan chains (GAG chains) exposes the stub epitope. Enzyme activity is detected by appearance of a dark smear. The antibody to NG2 detects both digested and undigested CSPGs. ChABC digestion results in a reduction of the high molecular weight smear as the GAG chains are removed. In the case of complete digestion a single band consisting of the core protein is observed. Signal sequences: MMP-2 = matrix metallo protease 2, GDNF = glial-derived neurotrophic factor, Ig Kappa = Ig kappa immunoglobulin light chain, PRL = prolactin, B sig = native bacterial signal sequence from the ChABC gene. C4 = unmodified bacterial ChABC.

### The 3’ UTR of the human β-actin gene targets ChABC to neuronal growth cones

We have shown previously that delivery of our modified ChABC via a lentiviral vector promotes sprouting and short-range regeneration of corticospinal axons *in vivo*, and that these beneficial effects are recapitulated when the same recombinant vector is used to deliver the enzyme in the vicinity of a spinal cord lesion [3]. Here we have extended these studies to determine if the efficacy of the modified enzyme can be enhanced further by targeting the enzyme to the neuronal growth cone. Sequences present in the 3’UTR of the β-actin mRNA are responsible for localising β-actin at neuronal growth cones [32]. We therefore first determined whether replacing the 3’UTR of the ChABC construct (bovine growth hormone poly A, from pcDNA3.1, Invitrogen) with that from the human β-actin gene is sufficient to target ChABC to the neuronal growth cone. This construct contains an MMP-2 signal sequence and all of the identified activity-enhancing mutations and codons optimized for expression in human cells. It also contains an mcherry tag to allow protein localisation to be determined. The efficiency of targeting was assessed by transfecting the different constructs into the neuronal cell line SH-SY-5Y. Substituting the 3’UTR from pcDNA 3.1 with that of human β-actin was sufficient to target ChABC to the growth cone. The positive control for targeting was the plasmid pCLNX GFP, a construct containing the same 3’UTR and known to target GFP to neuronal growth cones. This was compared to non-targeted GFP. Protein location within the cells was detected by immunohistochemistry and targeting was measured using the ratio of average growth cone fluorescence to average whole-cell fluorescence.

**Fig 3:**
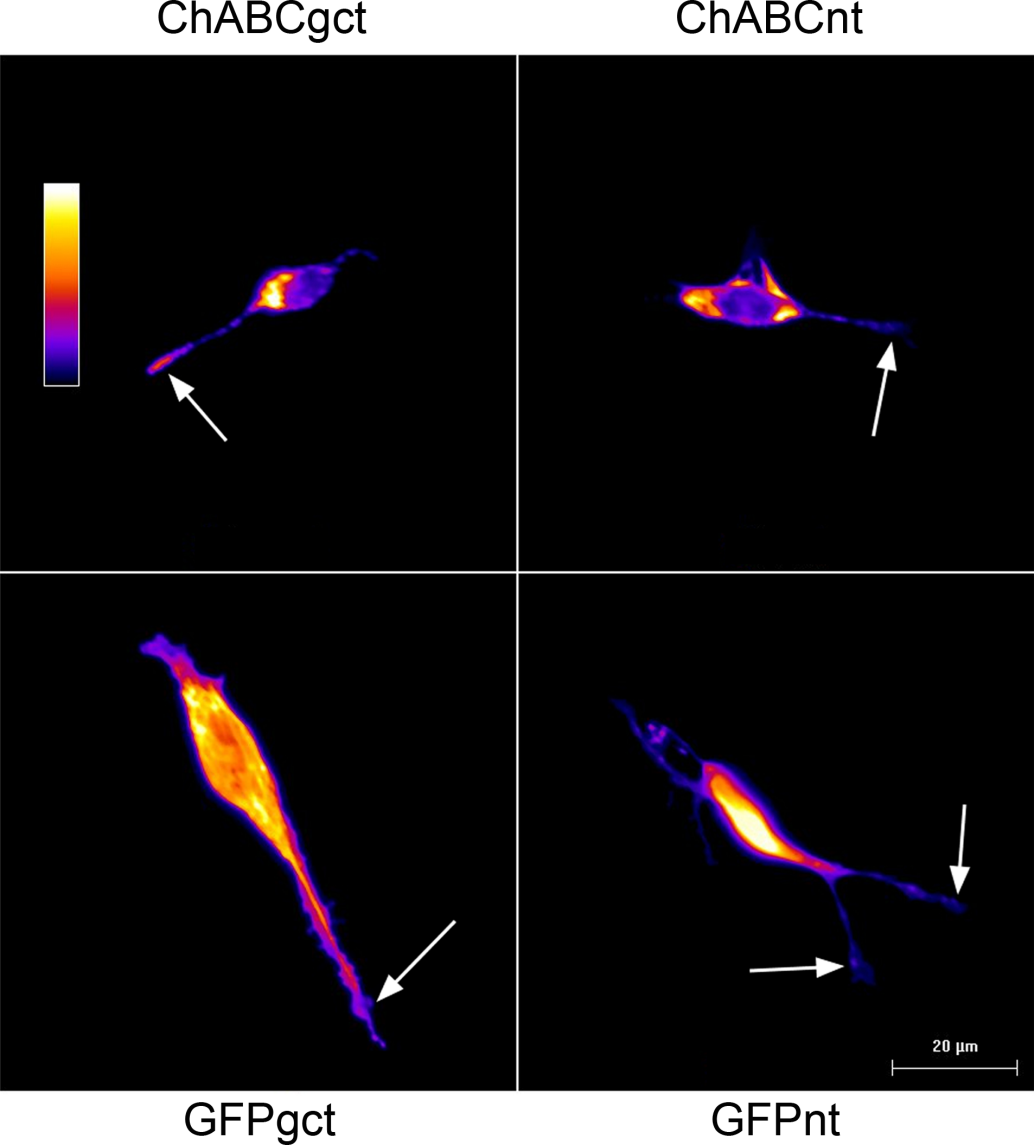
SHSY5Y neurons transfected with targeting or non-targeting constructs.

Addition of the 3’UTR to the ChABC cDNA resulted in highly significant growth cone targeting (P<0.001, n = 106 growth cones) of the enzyme. The positive control also targets GFP to the growth cone (P<0.029 n = 40) as expected. A typical image is shown in Fig 3. Whilst all the statistical data were gathered from experiments using SH-SY5Y neurons, we also observed similar targeting in NG108 cells, the cell line in which the growth cone targeting assay was first described [25] (S1 Fig).NG108 cells transfected with non-targeted or growt cone targeted constructs.

Enhanced staining for mcherry and GFP only at the neuronal growth cone of cells transfected with growth cone-targeting constructs is consistent with the local translation of ChABC and GFP mRNA at the growth cones of these neurons. Staining for the mcherry tag also shows that ChABC is present in the cell bodies of neurons transfected with both targeted and non-targeted constructs. This implies that the targeting efficiency is not 100%. However, a functional effect on neurite outgrowth is only observed in neurons (both SH-SY5Y cells and cortical neurons) transfected with ChABC-gct, see below. This suggests that the targeting event does not need to be complete to elicit a highly significant functional effect.

Top panel: growth-cone-targeted ChABC (ChABC-gct) & non-targeted ChABC (ChABC-nt). Bottom panel: Growth cone-targeted (GFP-gct) and non-targeted (GFP-nt) GFP. Pixel intensities were converted into false colour using image J to highlight regions of interest. False colour scale: white, maximum pixel intensity, black, minimum pixel intensity; arrows indicate growth cones where a higher level of pixel intensity is present in cells transfected with the targeting constructs.

When the relationship between growth cone:whole cell fluorescence ratio and whole-cell fluorescence was analysed post hoc, we found a significant negative correlation for cells transfected with the growth cone-targeted construct but not the non-targeted construct.

**Fig 4:**
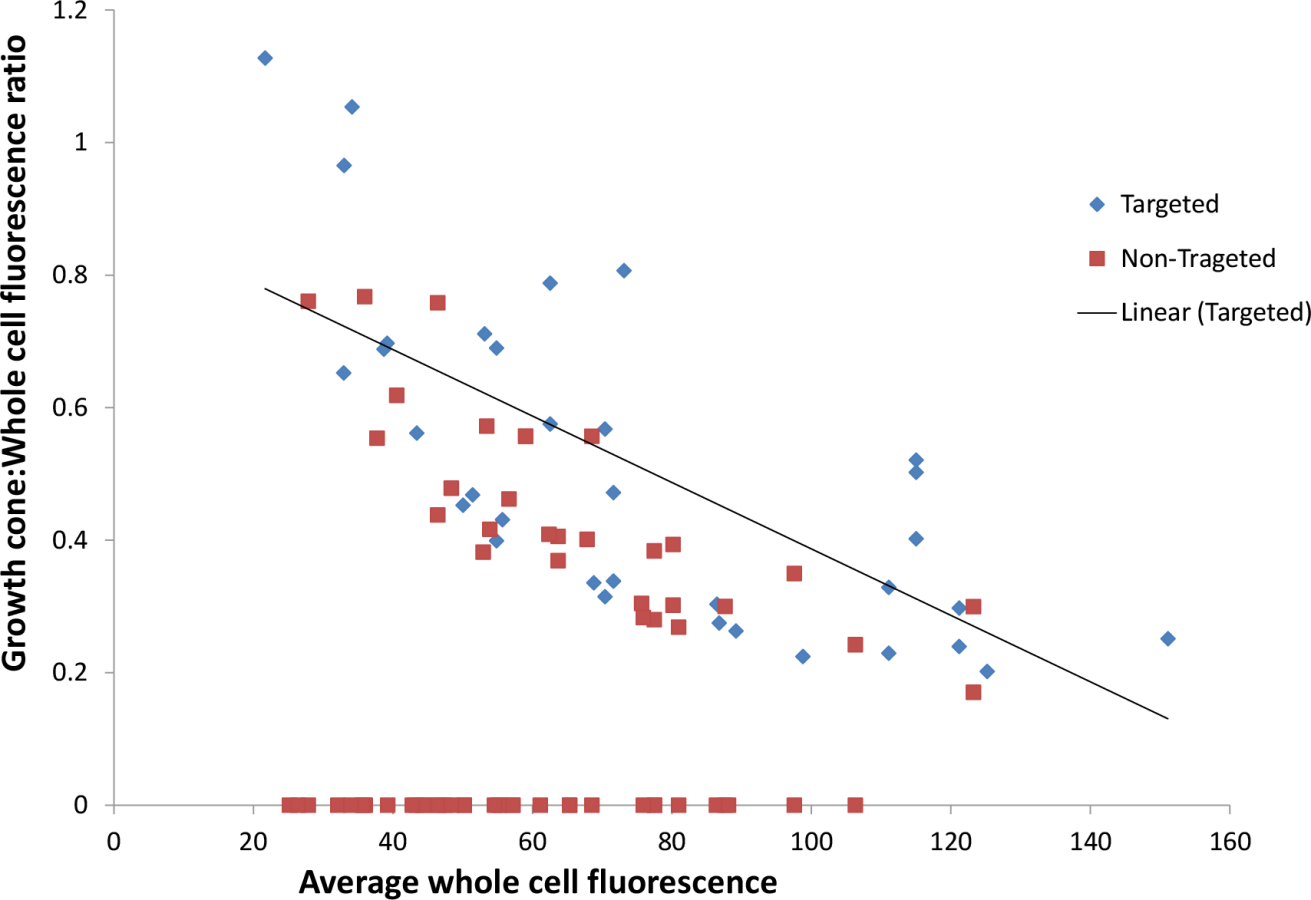
The relationship between the ratio of growth cone: Whole cell fluorescence and average whole cell fluorescence.

A linear regression line fitted for the targeted construct values shows a decrease in average growth cone/whole cell ratio as the average cell intensity increases (Fig 4). Thus when the transfection efficiency is high, targeting efficiency is reduced. It is also of note that cells exhibiting zero targeting were present in all groups. In the case of cells expressing the protein at very low levels, this could result from lack of detection, but a few cells exhibiting medium to high levels of whole-cell fluorescence also showed zero targeting. Thus, a minority of cells which were successfully transfected did not show active targeting. This was observed for both ChABC- and GFP-targeted constructs, suggesting it may be a general phenomenon.

Post hoc analysis (Pearson product moment correlation coefficient) showed extremely low levels of correlation for cells transfected with the non-targeted ChABC (r = -0.0067, n = 67) whilst we found a negative correlation for cells transfected with the growth cone-targeted ChABC (r = -0.5247, n = 39).

### Plating non-transfected cells onto a substrate containing chondroitin sulphate A (CSA) at 50μg/ml significantly inhibits neurite outgrowth

ChABC is known to promote axon outgrowth in an inhibitory environment [4,33]. We therefore established whether targeting ChABC to the neuronal growth cone further enhances this effect. We first determined the concentration of CSA required to produce a significant inhibition of neurite outgrowth from SH-SY5Y cells. A process extending from the cell which was longer than twice the diameter of the cell was considered to be a neurite. The 10 longest neurites from each neuron were measured and averaged. The average neurite length was then calculated from all neurons on each coverslip to give a final measure for each condition. Statistical analysis showed again that the data did not follow a normal distribution (p<0.001, n = 709 laminin, n = 232, CSA) and therefore the non-parametric MWU-test was used for comparisons. When neurons were plated onto a substrate containing CSA at 50μg/ml, a significant reduction in neurite length was seen compared to that obtained on laminin (Table 1) (P<0.001). This effect was reproducible between experiments (n = 6). These experiments were carried out using non-transfected cells but the results were confirmed in later experiments using cells transfected with the control construct encoding GFP.

**Table 1:** Comparisons (Mann Whitney U test) of neurite length of non-transfected neurons plated on to laminin or CSA.

Comparison	Median neurite length on laminin (μm)	n, laminin	Median neurite length on CSA (μm)	n, CSA
Neurite length on laminin vs CSA	21.9	709.0	12.0	232.0

Plating cells on to a substrate containing 50 μg/ml CSA significantly reduces neurite length. P < 0.001, (MWU test) n = number of neurites measured.

### Targeting ChABC to the neuronal growth cone increases neurite length and reverses the growth-inhibitory effect of CSA

To determine the effect of growth cone targeting on neurite outgrowth, neurons were transfected with targeted ChABC (ChABCgct), or non-targeted (ChABCnt) constructs and plated onto slides coated with laminin or laminin/CSA. Since targeting does not occur in all neurons transfected with the ChABCgct construct (see above), only those in which successful targeting had occurred were included in the analysis. These neurons showed a mean pixel intensity (growth cone: cell body) of 0.7 compared to the non-targeted ratio of 0.08.

When plated onto CSA-coated slides, neurons in which growth cone targeting of ChABC had occurred had significantly longer neurites than those transfected with the non-targeted construct n = 236 (Table 2). The median length of neurites from targeted neurons was twice that of neurons transfected with the non-targeted construct (P<0.001). Since neurons transfected with either the non-targeted or growth cone-targeted construct secrete similar amounts of enzyme (Morgan-Elson enzyme assay) this effect is consistent with enhanced secretion of ChABC from the growth cone. This supports the hypothesis that targeting ChABC to the growth cone has the potential to further enhance the efficacy of ChABC in promoting neurite outgrowth in an inhibitory environment. Neurons transfected with the GFP growth cone-targeted construct and grown on CSA did not show a similar increase in neurite length (data not shown), consistent with this being a ChABC-mediated effect. There was also no significant difference between neurite lengths of neurons transfected with the growth cone-targeted ChABC when grown on laminin or CSA, (Table 2) (P>0.05), suggesting that such targeting can successfully counteract the inhibitory effects of CSA.

**Table 2:** A comparison of neurite lengths from neurons (SH-SY5Y) transfected with the growth cone-targeting construct ChABCgct or the non-targeted construct ChABCnt on laminin or CSA.

Construct	Substrate	Median neurite length (μm)	n
GC targeted	Laminin	19.6	167
GC targeted	CSA	19.0	111
Non-targeted	Laminin	15.4	172
Non-targeted	CSA	9.5	69

Targeting ChABC to the growth cone significantly increases neurite outgrowth of neurons plated on to CSA, p < 0.001,(MWU test) This reverses the inhibition of neurite outgrowth seen on CSA using neurons transfected with the non-targeted construct; there is no significant difference in neurite length between cells transfected with ChABCgct plated on laminin compared to CSA, p > 0.05, (MWU test) n = 278. n = number of neurites measured.

### The growth promoting effects of growth cone-targeted ChABC are recapitulated in primary cultures of cortical neurons

Using the SH-SY5Y cell line we have demonstrated that the 3’UTR of β-actin targets ChABC to the neuronal growth cone and that this promotes neurite outgrowth in an inhibitory environment. To further investigate the potential efficacy of this new construct we extended this study to determine if these findings could be recapitulated in cultures of primary neurons transfected with the constructs. We used primary cultures of cortical neurons because they are particularly refractory to interventions designed to promote neurite outgrowth and they are a class of neuron that we want to target *in vivo* to promote regeneration following SCI. Cultures of cortical neurons were transfected the constructs encoding either growth cone-targeted ChABC or non- targeted ChABC. These neurons were then plated onto slides coated with CSA at a concentration previously established to be inhibitory to neurite outgrowth from these cells. Transfected cells were identified by staining the mcherry tag present on both constructs. In this experiment neurite lengths from all transfected cells were measured. The median neurite lengths of neurons transfected with non-targeted ChABC and plated onto CSA was 60.5μm. The median neurite length of neurons transfected with growth cone-targeted ChABC and plated onto CSA was 99.5μm, representing a 40% reduction of the inhibitory effect of CSA on neurite outgrowth, a value that is highly significant p<0.0001(MW U test) n = number of neurites measured (Table 3). This shows that targeting ChABC to the neuronal growth cone of CNS neurons also enhances their ability to extend neurites on an inhibitory substrate, confirming the robustness of this construct’s actions.

**Table 3:** A comparison of neurite lengths from cortical neurons transfected with the growth cone targeted (ChABC-gct) or non-targeted (ChABC-nt) constructs plated onto CSA.

Construct	Substrate	Median neurite length μm	n
ChABC-gct	CSA	99.5	90
ChABC-nt	CSA	60.5	90

Cortical neurons transfected with the construct encoding growth cone targeted had significantly longer neurites than those transfected with the construct encoding the non-targeted version. P<0.0001, (MWU test).

## Discussion

Many bacterial proteins have been expressed successfully in the cytoplasm of mammalian cells, for example the lacZ gene encoding beta-galactosidase and the diaminopimelic acid decarboxylase gene of *E*.*coli* [34]. Problems associated with expression of mammalian proteins in bacterial cells are also well documented, but very little has been documented regarding secretion of bacterial proteins by mammalian cells. We have previously identified the mammalian secretory pathway as a potential obstacle to successful secretion of bacterial proteins. In mammalian cells N-glycosylation plays a major role in the correct folding of proteins destined for the extracellular space, and misfolded proteins are eliminated [35]. Removal of selected N-glycosylation sites in the ChABC molecule allows secretion of active enzyme from mammalian cells [1]. The unexpected finding here that mutations N773Q and N856Q reduce secretion of ChABC enzyme suggests, conversely, that at least two sites on the ChABC protein need to be glycosylated for the enzyme to be secreted. This may be due to the large size of the protein, requiring carbohydrate chains at these positions to assist with its correct folding as it passes through the secretory pathway of a eukaryotic cell. Consistent with this, it has been shown that introduction of an N-glycosylation site increases secretion of heterologous proteins in yeast cells [36].

Of the few reports of successful secretion of bacterial enzymes by mammalian cells, a related enzyme, Chondroitinase AC from *Flavobacterium heparinium*, has been documented [37,38]. However this enzyme contains only four potential glycosylation sites, compared to seventeen present on ChABC, and only one site is in the vicinity of the enzyme's catalytic site. Additionally, *F*. *heparinium*, unlike most bacteria, is capable of post-translational glycosylation and native bacterial Chondroitinase AC is glycosylated [39]. Thus it is plausible that proteins from this bacterium, in common with those from archeabacteria, which also possess the ability to carry out post-translational glycosylation, have evolved to edit out cryptic signals for N-glycosylation at positions in the protein where such a modification would be detrimental. Indeed bacterial xylanase from *Clostridium Thermocellum* is efficiently synthesized and secreted by mammalian cells [40]. Nonetheless, an endo (1–4)-beta-glucanase gene C6.5 from *Bacillus subtilis* has been expressed in Chinese hamster ovary cells (CHO) and activity was 89% of the native enzyme. This protein, which contains only six potential N-glycosylation sites, was glycosylated by the CHO cells, but the sites that were glycosylated were at a considerable distance from the active site [41]. There are also two further reports of bacterial enzymes being successfully secreted by mammalian cells, *E*. *coli* chloramphenicol acetyl transferase [42] and *E*.*coli* maltose binding protein MalE [43]. It may be significant that both these proteins are significantly smaller than ChABC, 219 and 396 amino acids respectively compared to 1026 amino acids of ChABC, and are thus likely to undergo folding more easily. Moreover, consistent with our hypothesis that aberrant glycosylation can prevent secretion of bacterial proteins in an active form, both are secreted in a non-glycosylated form.

A growing number of reports are beginning to challenge the widely held view that signal sequences for secretion are functionally equivalent and largely interchangeable. There is now evidence that they have roles extending outside the regulation of initiation of translocation. Signal sequences have been shown to vary considerably in the efficiency with which they direct translocation of a secretory protein into the ER, with that from the secretory hormone prolactin (PRL) being one of the most efficient *in vitro* and *in vivo* [29]. Thus functions for the long-noted diversity in signal sequences are emerging, including influencing the efficiency of translocation and requirement for accessory components [28]. The hydrophobic domain has also been shown to influence at least two steps in maturation, this is the initiation of N-glycosylation and the timing of signal sequence cleavage [31]. Interestingly, the ChABC band on western blots migrates more slowly with the Ig kappa and GDNF signal sequences than with the MMP-2 signal sequence, which is consistent with an extra N-glycosylation site being occupied at N-7. This the only N-glycosylation site close enough to the N-terminus of the protein to be affected, since these signals direct earlier signal cleavage compared to the MMP-2 signal.

In view of these findings, we investigated the efficiency with which different signal sequences are able to direct secretion of ChABC from a range of cell types. We tested the prolactin sequence because it has been reported to be one of the most efficient at directing secretion [29], and the Ig Kappa signal sequence because it is included on most commercially available eukaryotic expression vectors and therefore considered a generally efficient signal. We also tested the GDNF and MMP-2 signal sequences because these proteins are secreted in the CNS, where we ultimately want to express ChABC, as well as the native bacterial signal sequence. We also assessed their function in a range of cell types, including the glial cell lines Neu7 [23] and SCTM41 [22], as well as primary cultures of glia, being the CNS resident cells that we ultimately wish to target. We found that the MMP-2, GDNF and Ig kappa signals function with the ChABC coding sequence, but that the efficiency with which these signals direct secretion from cells varies and is cell-specific. The MMP-2 signal sequence was found to function most efficiently overall, and we have now demonstrated that it functions in neurons *in vivo* [44]. The prolactin and native bacterial signal sequences were not effective in the cell lines tested. The prolactin signal sequence has been reported to be effective in Cos7 cells [29], and our observation that it failed to direct secretion of ChABC from the cell types tested in this study, including Cos7 cells, suggests that there is a substrate-specific component affecting the efficiency of secretion from the different signal sequences, a phenomenon that has been reported previously [29].

It has also been noted for mammalian proteins that the most effective signal sequence for a protein is its native signal [30]. However our study suggests that this may not apply to a bacterial protein expressed in a eukaryotic cell. We found that the native bacterial signal sequence for ChABC functioned poorly, if at all, in mammalian cells compared to those derived from mammalian proteins. The wide variation in signal sequence efficiency between cell types has also been reported for mammalian proteins [29], but the MMP-2 signal functioned efficiently in all our cell types tested. We have also shown that it functions effectively in neurons and glia *in vivo* [3,5]. In sum, our findings are in agreement with reports by others and demonstrate the importance of testing several signal sequences in a range of cell types when optimising secretion of transgene products, especially those of non-mammalian origin.

We further extended the study of ChABC trafficking in mammalian cells to determine whether we could direct the enzyme to the neuronal growth cone. We wanted to assess whether targeting ChABC to the growth cone was more efficacious in promoting axon growth on an inhibitory substrate than non-localized expression. Our approach was to replace the 3’UTR of bovine growth hormone on the transgene with that of β-actin. Attaching the 3’UTR from β-actin is sufficient to target GFP to neuronal growth cones [32], and we have shown, using the SH-SY5Y neuronal cell line, that this is also sufficient to enhance the levels of the ChABC mCherry fusion protein at the growth cone. However, the efficiency of targeting is not 100% and decreases when the enzyme is expressed at high levels. This may be due to saturation of the machinery responsible for trafficking mRNAs in the axon, a phenomenon that has been reported before [45]. Nonetheless, neurites extending from SH-SY-5Y neurons transfected with the growth cone-targeted chondroitinase construct were almost twice as long on the inhibitory CSA substrate compared to neurites from neurons transfected with the non-targeted construct. An enhancement of neurite outgrowth is also seen when cortical neurons are transfected with ChABC-gct and plated onto CSA. This suggests that focusing digestion of CSA to the neurite tip is more effective at promoting neurite outgrowth than generalised secretion of the enzyme from the cell as a whole. Thus ChABCgct is likely to be more efficacious at promoting neurite elongation following SCI.

In conclusion, we have shown that variations in both glycosylation and signal sequence influence the efficiency of ChABC secretion from mammalian cells. We have further shown that targeting ChABC to the growth cone increases the length of neurites grown in an environment inhibitory compared to those from neurons transfected with non-targeted ChABC. Such targeting may provide a powerful way of promoting axon outgrowth and functional recovery following SCI.

## Supporting information

S1 Fig NG108 cells transfected with growth cone- targeting or non-targeting constructs. (TIF)

S1 Table Sequences of the junctions between the different signal sequences and the 5’ end of the ChABC gene. (DOCX)
