# Supplementary figures and images for: Trafficking and processing of bacterial proteins by mammalian cells: Insights from chondroitinase ABC
Source: PLoS One. 2017 Nov 9;12(11):e0186759. doi: 10.1371/journal.pone.0186759 (PMC5679598; doi:10.1371/journal.pone.0186759)

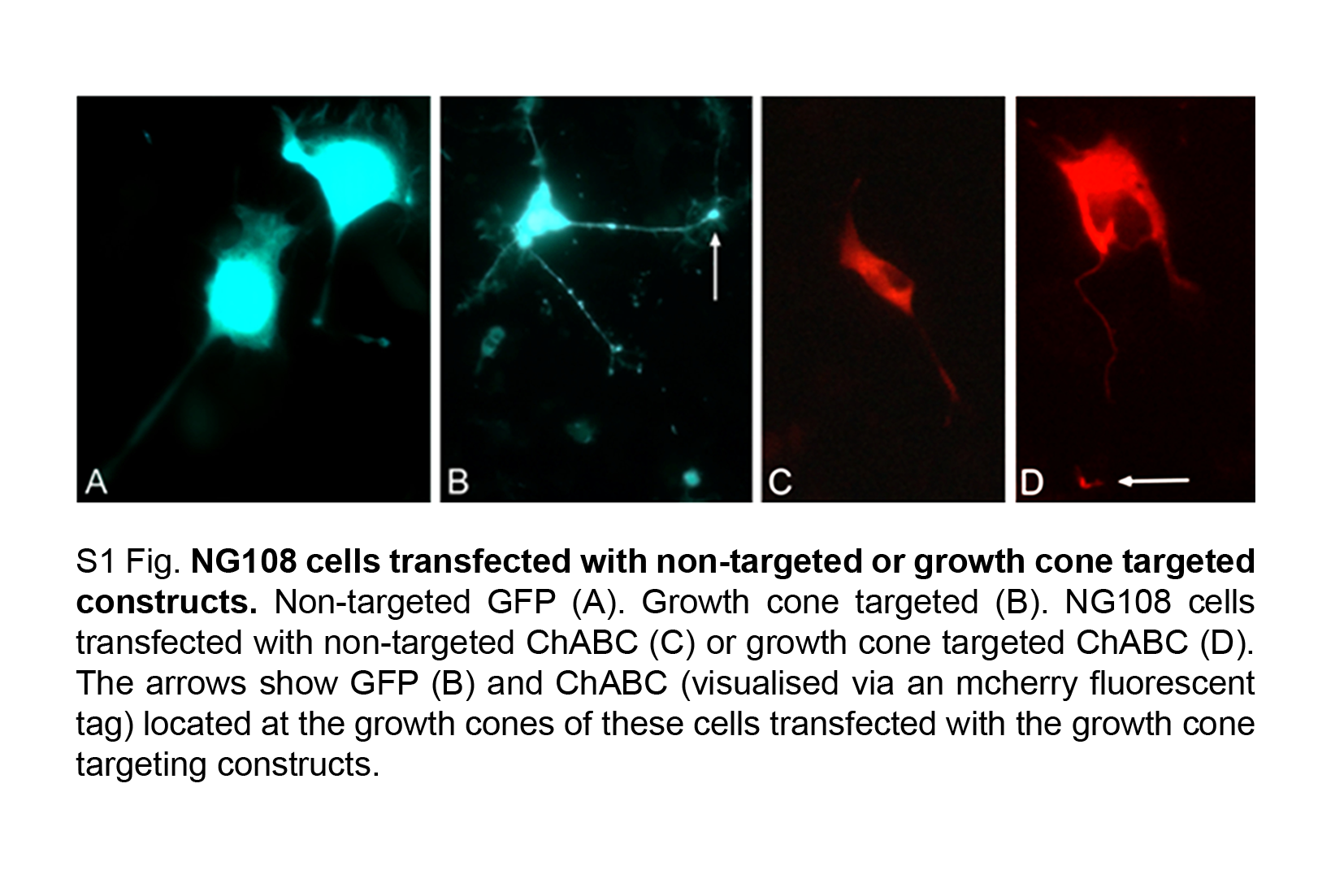

Supplement: S1 Fig — (TIF) [file pone.0186759.s001.tif]
