# Supplementary material for: Trafficking and processing of bacterial proteins by mammalian cells: Insights from chondroitinase ABC
Source: PLoS One. 2017 Nov 9;12(11):e0186759. doi: 10.1371/journal.pone.0186759 (PMC5679598; doi:10.1371/journal.pone.0186759)
Supplement: S1 Table — (DOCX) [file pone.0186759.s002.docx]

**S1 Table**

| **Signal sequences: Junction sequences between signal and ChABC gene** | | | | | | |
| --- | --- | --- | --- | --- | --- | --- |
|  | | | | | | |
|  | PRL | |  | SpeI | |  |
| Prolactin | GTG | AGC | GCA | ACT | AGT |  |
|  | V | S | A | T | S |  |
|  |  |  | ChABC | | |  |
|  | | | | | |  |
|  | MMP-2 | |  |  |  |  |
| MMP-2 | ATC | GCC | GCC | ACT | AGT |  |
|  | I | A | A | T | S |  |
|  | | | | | |  |
|  | Bsig | |  |  |  |  |
| Native bacterial | CTG | GCC | GCA | ACT | AGT |  |
|  | L | A | A | T | S |  |
|  | | | | | |  |
|  | Igκ | |  |  |  |  |
| Ig kappa | GGT | GAG | GCA | ACT | AGT |  |
|  | G | D | A | T | S |  |
|  | | | | | |  |
|  | GDNF | |  |  |  |  |
| GDNF | TCT | GCC | GCC | ACT | AGT |  |
|  | S | A | A | T | S |  |
